# Effects of simultaneous use of m-NMES and language training on brain functional connectivity in stroke patients with aphasia: A randomized controlled clinical trial

**DOI:** 10.3389/fnagi.2022.965486

**Published:** 2022-09-07

**Authors:** Hui Xie, Jing Jing, Yanping Ma, Ying Song, Jiahui Yin, Gongcheng Xu, Xinglou Li, Zengyong Li, Yonghui Wang

**Affiliations:** ^1^Rehabilitation Center, Qilu Hospital of Shandong University, Jinan, Shandong, China; ^2^Beijing Key Laboratory of Rehabilitation Technical Aids for Old-Age Disability, National Research Center for Rehabilitation Technical Aids, Beijing, China; ^3^Key Laboratory for Biomechanics and Mechanobiology of Ministry of Education, School of Biological Science and Medical Engineering, Beihang University, Beijing, China; ^4^Key Laboratory of Rehabilitation Aids Technology and System of the Ministry of Civil Affairs, Beijing, China

**Keywords:** aphasia, neuromuscular electrical stimulation on median nerve, language training, brain functional connectivity, effect of rehabilitation

## Abstract

**Introduction:**

The m-NMES had been demonstrated to redistribute brain resources and induce plastic changes in the stroke patients. However, the physiological mechanism and clinical efficacy of m-NMES combination with existing clinical rehabilitation programs remains unclear in patients with aphasia after stroke. This study aimed to investigate the effects of simultaneous use of m-NMES and language training (m-NMES-LT) with on cerebral oscillations and brain connection, as well as the effect on clinical efficacy.

**Materials and methods:**

Total 21 right–handed adult patients with aphasia were randomly assigned to language training (LT) group and m-NMES-LT group, and tissue concentration of oxyhemoglobin and deoxyhemoglobin oscillations were measured by functional near-infrared spectroscopy in resting and treatment state during three consecutive weeks. Five characteristic frequency signals (I, 0.6–2 Hz; II, 0.145–0.6 Hz; III, 0.052–0.145 Hz; IV, 0.021–0.052 Hz; and V, 0.0095–0.021 Hz) were identified using the wavelet method. The wavelet amplitude (WA) and wavelet phase coherence (WPCO) were calculated to describe the frequency-specific cortical activities.

**Results:**

The m-NMES-LT induced significantly higher WA values in contralesional PFC in intervals I, II, and V, and ipsilesional MC in intervals I-V than the resting state. The WPCO values between ipsilesional PFC-MC in interval III-IV, and between bilateral MC in interval III-IV were significantly higher than resting state. In addition, there was a significant positive correlation between WPCO and Western Aphasia Battery in m-NMES-LT group.

**Conclusion:**

The language training combined with neuromuscular electrical stimulation on median nerve could improve and achieve higher clinical efficacy for aphasia. This is attributed to the m-NMES-LT could enhance cortical activation and brain functional connectivity in patients with aphasia, which was derived from myogenic, neurogenic, and endothelial cell metabolic activities.

## Introduction

As a neurological disease, stroke has the characteristics of high morbidity, mortality, and disability rate (Feigin et al., 2014). Aphasia is one of the most common complications and sequelae of stroke and it was often accompanied by a reduction of available vocabulary, linguistic rules, and verbal retention span, as well as impaired comprehension and production of messages, which accounts for up to 20–30% of the stroke disability rate. The characteristics of aphasia interfere with the patient’s normal communication with the external environment, thus seriously affecting the patients’ emotions, family, and social life. Therefore, understanding and strengthening effective rehabilitation treatment after stroke is of great significance for related functional recovery in aphasia patients.

Traditional language training (LT) for aphasia, such as schuell stimulation, was the most widely used treatment for aphasia in clinical practice (Seniów et al., 2013). Schuell stimulation promoted the reconstruction and recovery of the devalued speech symbol system in stroke patients with aphasia to the greatest extent by using strong and controlled auditory stimulation. According to the degree and type of aphasia, it repeatedly used topics that were easily accepted by the patient, and give the patient corresponding auditory training stimulation. It could obtain optimistic effect in the early recovery of stroke. Studies had shown that auditory sensory input could alter the brain electrical activity, increasing the frequency of firing of neurons and the number of fibers activated and influence the structure and function of the brain, which is the mechanism of LT. However, LT required intensive and repetitive auditory stimulation to achieve the recovery of patients with aphasia, and was not sensitive to severe patients (Chapey, 2012; Coelho et al., 2012). Therefore, it is urgent to find a more efficient rehabilitation method of aphasia patients in recovery period after stroke.

Neuromuscular electrical stimulation on median nerve (m-NMES) was a commonly utilized tool to augment peripheral nerve activation. Conventional m-NMES placed electrodes on the skin of 2 cm above the wrist stripes to deliver electrical stimulation. Because it occupied a large area in the centrally innervated area and was the peripheral portal of the central nervous system, the electrical stimulation signals acting on the median nerve could be projected in a large range in the cerebral cortex and had a significant therapeutic effect. Neuroimaging studies had indicated that m-NMES could improve cortical activities and reorganization for a period of time after sensory input in the form of somatosensory electrical stimulation, which demonstrated the potential of m-NMES to redistribute brain resources and induce plastic changes in the stroke patients (Conforto et al., 2007, 2010). Our previous studies had shown that m-NMES could trigger sensorimotor stimulations of the affected hand that sequentially induced neural plasticity and involved functional reorganization of distant cortical areas in patients with aphasia after stroke (Huo et al., 2019). Therefore, we hypothesize that simultaneous use of m-NMES and LT (m-NMES-LT) in patients with aphasia may better induce the activation of the cerebral cortex and the reorganization of the brain network, thereby effectively improving the clinical efficacy of aphasia rehabilitation. The research based on neurophysiological measurements could help us better understand the underlying physiological mechanisms of this model.

The fNIRS is a new non-invasive imaging method of brain function that based on the neuro-metabolic coupling mechanism and the neuro-vascular coupling mechanism. It can indirectly reflect the activity of cerebral cortex and brain functional network (Lloyd-Fox et al., 2010; Scholkmann et al., 2014). The technology has good temporal and spatial resolution and can be realized real-time, non-invasive, continuous blood oxygen saturation monitoring (Jobsis, 1977; Naseer and Hong, 2015). Thus, it is readily applicable in clinical settings to detect hemodynamic fluctuation in different states of stroke patients, and it has been widely used in brain function evaluation, cognitive science and other fields (Tachtsidis and Papaioannou, 2013; Pinti et al., 2020). Studies have shown that the prefrontal cortex (PFC) plays an important role in language function. Language activity could cause a multiplicity of language-activated cerebral blood oxygenation and hemodynamic changes in PFC (Binder et al., 1997; Sakatani et al., 1998; Cotelli et al., 2012). The activation of the motor cortex (MC) was tightly connected to the language system (Pulvermüller and Fadiga, 2010; Meinzer et al., 2011, 2016), which could facilitate language processing in patients with aphasia (Meinzer et al., 2011; Harnish et al., 2014), and the occipital cortex (OC) had important value in the study of aphasia (Nelles et al., 1998; Szaflarski et al., 2011; Garcia-Azorin et al., 2014). Therefore, we tried to use fNIRS to detected the changes in delta [O_2_Hb] and [HHb] of PFC, MC, and OC.

Current models suggest that language functions result from functional interactions of distant temporal, frontal and parietal brain regions within a left-lateralized network (Hickok and Poeppel, 2007; Friederici, 2012; Gh and Ds, 2019). So, we choosed subjects with left stroke in this study and used fNIRS technology as the monitoring means, aimed to investigate the changes of m-NMES-LT on cerebral oscillations and brain functional reorganization. The results may help broaden the understanding of the contribution of aphasia recovery.

## Materials and methods

This study was a randomized, two-arm trial. The experimental procedures were approved by the Human Ethics Committee of National Research Center for Rehabilitation Technical Aids and were in accordance with the ethical standards specified by the Helsinki Declaration of 1975 (revised in 2008). It has been registered with the Chinese Clinical Trials Registry website under the trial number ChiCTR2100048433. Rehabilitation Center, Qilu Hospital of Shandong University and the Human Ethics Committee of National Research Center for Rehabilitation Technical Aids undertook this project.

### Participants

A total of 21 right-handed adult patients with aphasia after stroke were finally recruited to participate and completed this study through inpatient Qilu Hospital of Shandong University. All patients were sTables–ever after a first or recurrent stroke. The inclusion criteria were as follows: (1) left hemisphere lesion; (2) those with language functional impairment after stroke; (3) there is a need for rehabilitation. The exclusion criteria were as follows: (1) left-handed; (2) extremity dermatitis; (3) history of neurological, psychiatric, or auditory symptoms; (4) clinically significant or unstable medical disorder; (5) with brain trauma or had undergone brain surgery; (6) smoking and drinking. The researchers introduced the basic information (including experimental purposes, procedures, schedules, announcements, and contributions) of the experiment to the study participants and obtained their consent.

### Study design and randomization

**FIGURE 1:**
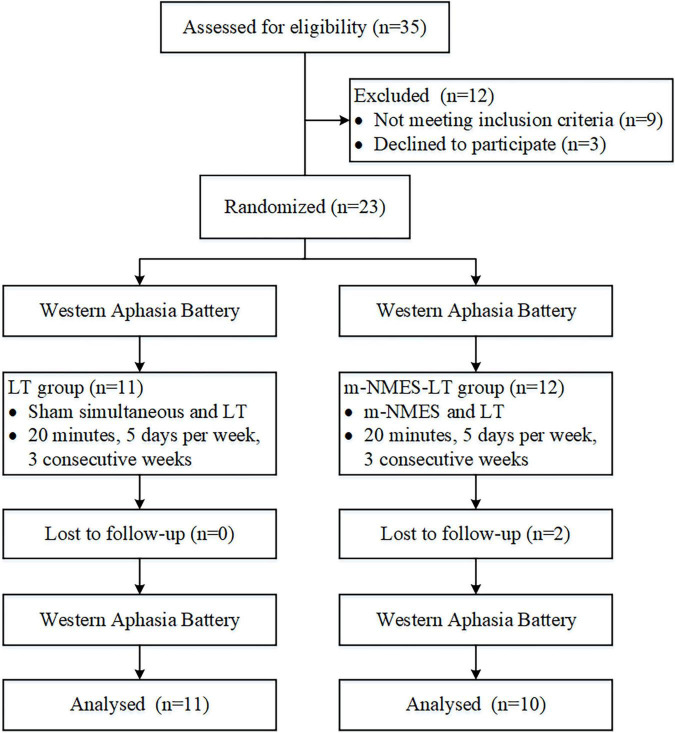
The enrollment and allocation of participants.

The sample size calculation was based on the previous studies (Mattioli et al., 2013). The total sample of subjects achieves 80% power to detect differences among the means versus the alternative of equal means using an *F* test with a 0.05 significance level. The minimum sample size was 20. Participants were randomly allocated to one of two groups: the LT group (namely simultaneous use of language training therapy and sham intervention) and the m-NMES-LT group (namely simultaneous use of language training therapy and m-NMES). Computer-generated numbers were used to randomly assign these patients. Opaque, sealed envelopes were used to conceal patient allocation and patients were allocated to the groups at random and patients were blinded after assignment to interventions. The enrollment and allocation of participants illustrated that 35 participants were assessed for eligibility in the study, nine participants withdrew from the study because of not meeting the inclusion criteria, three participants or families refused to consent to this study, and no participant was unable to be assessed. Two participants were excluded dropped out of the follow-up study because of loose detectors, as shown as Figure 1. Each post stroke aphasic patient was treated 5°days per week for three consecutive weeks, and underwent the Western Aphasia Battery (WAB) immediately when they were admitted to the hospital and after the 3°weeks of treatment. The fNIRS was implemented continuously throughout the experiment. The study was conducted from 6 July 2021 to 30 Dec 2021 at the Rehabilitation Center, Qilu Hospital of Shandong University.

### Procedures

The experiment was divided into resting state and treatment state in both LT group and m-NMES-LT group. The process of study took place in the Speech and Language Therapy Room at the hospital. Both groups of patients received the same type of LT. The task of LT was chosen and implemented by an experienced speech and language therapist, and included such domains as listening comprehension, repetition, word, and picture matching, verbal expression and conversational speech. Although the general nature of LT was the same for all patients, the set of tasks was matched to the type and severity of the individual aphasic symptoms. The m-NMES (rectangular pulse, 50 Hz) was delivered with a 40-s on−20-s off cycle. The intensity was set at the highest tolerable level of participants and sufficient to create a visible muscle twitch or contraction without pain.

Before the experiment, all participants were required to sit for 5–10 min in a noiseless environment to eliminate existing hemodynamic reactions induced by their activity. Subsequently, the fNIRS was worn by scientific assistant for the participants, and the real-time detection (in clouding resting and treatment state) was performed. One thing to note about the resting state is that during the resting state, patients were instructed to stay awake with their eyes closed and remain quiet for 10 min. After the resting state, the professional therapist wears the m-NMES equipment for the participants in both LT and m-NMES-LT groups. In the treatment state, the m-NMES-LT group received weak electric shocks were applied to the right wrist above the median nerve in, and the LT group received sham stimulation at the same sites in the same order but no treatment or electrical stimulation dose was applied. Patients in each group were treated once per day for 20 min, 5°days per week (Monday to Friday), for three consecutive weeks. The staff recorded the basic information of the participants on the day before the experiment.

### Functional near–infrared spectroscopy measurement

**FIGURE 2:**
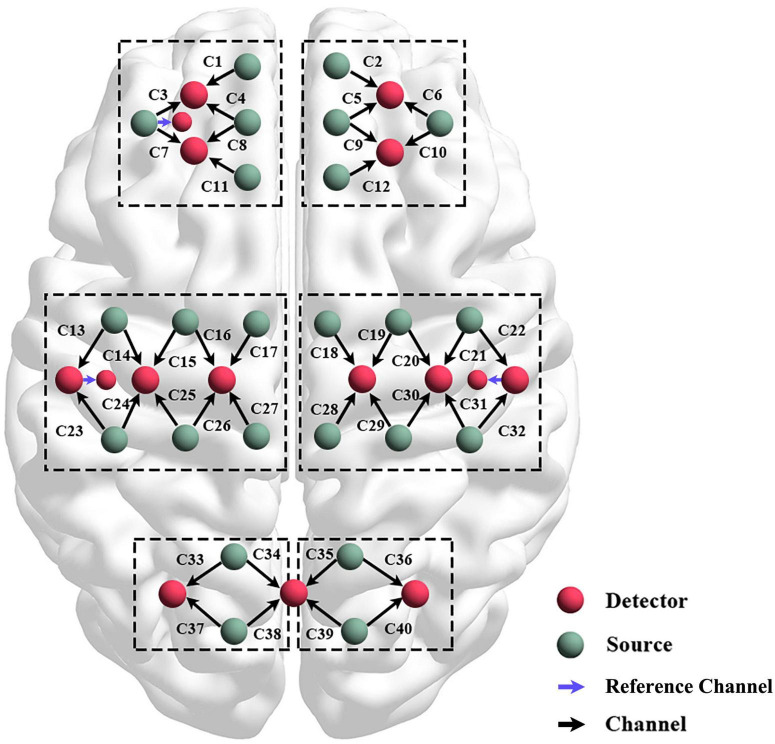
Schematic diagram of the experimental setup. Configuration of 24 source optodes, 16 detector optodes, and 43 measurement channels.

A technology multi–channel tissue oxygenation monitor with continuous–wave (NirSmart, Danyang Huichuang Medical Equipment Co., Ltd., Beijing, China) with wavelength of 740 and 850 nm was used in fNIRS measurements. To ensure that infrared light could pass through the skull to the gray matter, each sensor of the instrument consisted of a light emitting diode and a detector optode with a distance of 30 mm. We initially set all differential path–length factors to 7.0, and the sampling rate was 10 Hz. The calibration function of the instrument and the corresponding template were used to ascertain the channels to fill exactly in correspondence of the 10/10 electrode positions according to different head sizes. Each optode was connected to the scalp surface by using a customized hard plastic cap, and covered with a black cloth to prevent the penetration of the environmental light. In this study, the fNIRS channels were defined as the middle point of the corresponding light source-detector pairs. A total of 43 measurement channels, including 24 light source probes, and 16 detector probes, were symmetrically positioned over the regions of the left (ipsilesional) and right (contralesional) PFCs (LPFC/RPFC), MCs (LMC/RMC), and OCs (LOC/ROC) as shown in Figure 2.

### Data pre–processing

The fNIRS data were preprocessed using the NIRS Toolbox in MATLAB R2020a (MathWorks Inc., Natick, MA, United States). The pre–processing method of fNIRS data had been elaborated in our previous studies (Tan et al., 2015, 2016; Xu et al., 2017; Xie et al., 2019). Simply put, a moving average filter was used to eliminate the obvious abnormal points in the signal and the artifact portion was removed by cubic spline interpolation. Then independent component analysis was performed on delta [O_2_Hb] and [HHb] signals of each channel to determine the components that might be related to noise and artifacts, and three reference channels with 10 mm source–detector distance were used to extract scalp blood flow changes on signals. Finally, we retained the 0.0095–2 Hz portion of the filtering signal obtained using the six–order Butterworth band–pass filter.

### Wavelet transform

Continuous wavelet transform (CWT) is a wavelet analysis method used to analyze near-infrared brain oxygen signal, which has good time domain and frequency domain, and can analyze signal characteristics in time domain and frequency domain simultaneously. In recent years, this method has been applied to the field of biomedical engineering to analyze the interaction between various physiological parameters of human body. CWT could project time series from time domain to frequency domain and enable us to continuously derive the frequency content in time by adjusting the length of wavelet windows. Since the Morlet wavelet could locate independent time and meet the requirements of frequency resolution, the Morlet wavelet was used as the mother wavelet for wavelet transform.

In the spontaneous cerebral oxygen signal, the specific frequency interval distinguished by the wavelet transform had different physiological sources. Five frequency intervals corresponding to different physiological sources have been identified in our previous studies (Li et al., 2010; Bu et al., 2017): 0.6–2 Hz, synchronization of cardiac (I); 0.145–0.6 Hz, respiratory (II); 0.052–0.145 Hz, myogenic (III); 0.021–0.052 Hz, neurogenic (IV); 0.0095–0.021 Hz, endothelial cell metabolic (V). It can also be interpreted as the frequency intervals I and II reflect systemic regulation activities, whereas frequency intervals III–IV indicate neurovascular coupling. These can reflect the physiological source of cerebral activation and functional connectivity changes, which may be helpful to understand the neural mechanisms of brain plasticity and reorganization of the network after therapy in patients with aphasia and provide new ideas and methods for understanding the mechanism of brain function rehabilitation.

### Wavelet amplitude

The results of wavelet transform were averaged over the time domain to obtain the wavelet amplitude (WA) of each Δ [O_2_Hb] and [HHb] signal at each time and frequency, which reflects the magnitude of the fluctuation of the original signal at a certain frequency. WA of the Δ [O_2_Hb] and [HHb] signal represents the changes of regional cerebral blood flow with the activity of cerebral cortex during different conditions. Functional hyperaemia or neurovascular coupling could increase regional cerebral blood flow by activating local neurons to match the needs blood and nutrients of local brain cells in the task state (Willie et al., 2014). Thus, WA is characterized by the intensity or activation of the cerebral cortex.

### Wavelet phase coherence

The FC was calculated using wavelet phase coherence (WPCO), which is a method of using the phase information of the signal to evaluate the correlation of two signals. It could analyze the correlation of multiple different time series, observe their relationship in different frequency and time, and then put forward the appropriate interpretation mechanism. Some academics had made this method been applied to medicine, to analyze the relationship between the various physiological signals. The WPCO value was between 0 and 1, and the value quantitatively represents the instantaneous phase of the two signals at a consistent degree throughout the continuous process of the time series to identify possible connectivity (Bernjak et al., 2012). The high WPCO value indicates that an agreement between the two cortical regions exists, otherwise it indicates that weaker relationships between the two delta signals exist (Han et al., 2014).

### Statistical analysis

The Kolmogorov–Smirnov test and Levene test of participant’s data were performed to ensure that the assumptions required for parameter analysis were satisfied. The Pearson’s correlation was used for correlation analysis between WAB and spontaneous cerebral oxygen signal. The one–way ANOVA was performed on the region–wise WA and WPCO. The Bonferroni correction was used for the multiple comparisons. Totally there were three inter-groups pair-wise comparisons (resting state VS LT state, resting state VS m-NMES-LT state, and LT state VS. m-NMES-LT state), thereby the corrected *p*-value threshold was set at *p* < 0.0167.

## Results

### Demographic characteristic

Table 1 shows the demographic characteristic and WAB scores. There were no significant differences in baseline demographic or clinical features between the groups. Similarly, there were no significant group differences in any of the preintervention outcome measures as shown as in Table 2.

**TABLE 1:** Characteristics of participants with aphasia.

Patients	Group	Sex	Age	Etiology	Site of lesion	Time (month)	Aphasia type	WAB (Pre/Post 3°weeks treatment)				
								
								SS	AC	RT	NM	AQ
Pt 1	LT	F	57	Hemorrhage	Basal ganglia	0.9	Broca	4/7	6.8/7.2	0.6/2.5	0.9/4.2	24.5/41.8
Pt 2	LT	F	55	Hemorrhage	Basal ganglia	4.77	Transcortical mixed	7/12	3.9/6.3	6.9/7.4	4.4/6.3	44.3/63.9
Pt 3	LT	F	68	Infarction	Basal ganglia	0.5	Wernicke	10/12	4.9/5.8	6.9/7.5	4.1/4	51.8/58.6
Pt 4	LT	M	46	Hemorrhage	Basal ganglia	9.03	Global	0/1	3.2/4.6	0/0	0/0	6.4/11.2
Pt 5	LT	M	48	Infarction	FTP	2.03	Global	0/0	0.9/0.3	0/0	0/0	1.8/0.6
Pt 6	LT	M	58	Infarction	MCA	0.77	Global	0/2	1.1/2.4	0/0.9	0/0	2.2/10.6
Pt 7	LT	M	75	Hemorrhage	Basal ganglia	4.2	Global	0/4	1.1/1.6	2.1/2.6	0/0	6.4/16.4
Pt 8	LT	M	60	Infarction	FTP	3.13	Global	0/3	0.2/2.7	2.6/4	0/0.6	5.6/20.6
Pt 9	LT	M	49	Infarction	Basal ganglia	1.37	Transcortical motor	0/4	3.6/3.9	0/1	0/0	7.2/17.8
Pt 10	LT	M	46	Hemorrhage	MCA	0.4	Transcortical sensory	0/4	0.2/2.4	0/1.1	0/0	12.4/21
Pt 11	LT	M	41	Infarction	Basal ganglia	2.07	Transcortical mixed	6/7	2.4/2.4	4.1/5	2.7/3	24.4/34.8
Pt 12	m-NMES-LT	M	44	Infarction	MCA	4	Global	0/4	0.4/2.4	0/0.2	0/0	0.7/13.1
Pt 13	m-NMES-LT	M	59	Infarction	FTP	1.67	Global	1/3	0.5/1.7	0/0.8	0/0	3/11
Pt 14	m-NMES-LT	M	79	Infarction	Basal ganglia	2	Global	0/3	0.1/1.1	0/1	0/0	0.2/10.2
Pt 15	m-NMES-LT	F	65	Infarction	Basal ganglia	4.33	Global	1/4	1/2.5	0/3	0/0.6	4/20.2
Pt 16	m-NMES-LT	M	63	Infarction	MCA	0.67	Global	1/4	0.3/2.4	0/1.4	0/0	2.6/15.6
Pt 17	m-NMES-LT	M	47	Infarction	MCA	3	Global	4/9	3.8/3.7	0.8/2.5	3.1/3.8	23.4/38
Pt 18	m-NMES-LT	M	57	Infarction	ITP	0.67	Broca	11/15	6.6/7.9	0.8/6.2	4.8/8	46.4/74.2
Pt 19	m-NMES-LT	F	50	Infarction	MCA	0.83	Transcortical mixed	7/9	2.9/3.5	5.2/7.2	0.2/1	30.6/41.4
Pt 20	m-NMES-LT	M	64	Infarction	MCA	2.43	Global	0/9	0.2/4.3	0.1/2.1	0/4.1	0.6/39
Pt 21	m-NMES-LT	F	47	Infarction	Basal ganglia	4.83	Broca	4/4	4.1/4.8	2/3.4	0.3/0.6	20.8/25.6

Pt, Patient; M, Male; F, Female; MCA, Middle cerebral artery; FTP, Frontotemporal-parietal; ITP, Insula-temporoparietal; WAB, Western Aphasia Battery; SS, Spontaneous speech; AC, Auditory comprehension; RT, Repetition; NM, Naming; AQ, Aphasia quotient.

**TABLE 2:** Demographic characteristics: Mean (*SD*).

Demographic characteristics	LT group (*n*=11)	m-NMES-LT group (*n*=10)	*P-value*
Age (Years), mean (*SD*)	54.82 (± 10.25)	57.50 (± 10.81)	0.566
Male/female (N)	8/3	7/3	0.890
Time (Month)	2.65 (± 2.58)	2.44 (± 1.55)	0.827
WAB (Per 3°weeks treatment)			
Spontaneous speech	2.45 (± 3.67)	2.9 (± 3.66)	0.784
Auditory comprehension	2.57 (± 2.12)	1.99 (± 2.24)	0.547
Repetition	2.11 (± 2.73)	0.89 (± 1.65)	0.237
Naming	1.10 (± 1.76)	0.84 (± 1.69)	0.734
Aphasia quotient	17.00 (± 17.29)	13.23 (± 16.17)	0.613

### Cerebral activation

As an indication of cerebral activation, WA values at 0.0095–2°Hz frequency intervals in the two groups showed specific differences in different brain regions. Our cortical activation results showed that, from the perspective of neurovascular coupling, RPFC seemed to be more sensitive to LT, while LMC was more active under m-NMES-LT treatment. However, there was no evidence that the WA values showed differences in LT and m-NMES-LT treatment in LPFC or RMC.

**FIGURE 3:**
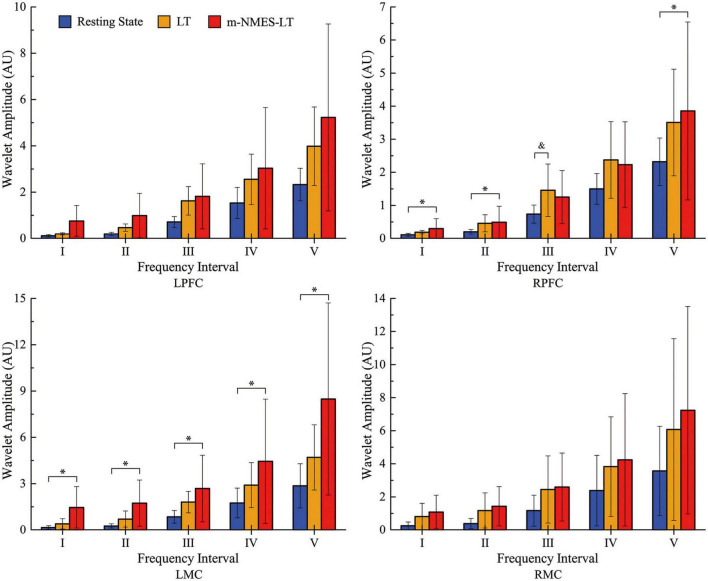
Wavelet amplitude (WA) data between resting and treatment states: Mean (SD). *(*p* < 0.05) indicate significant difference between the resting state and m-NMES-LT state. &(*p* < 0.05) indicate significant difference between the resting state and LT state. L, left; R, right; MC, motor cortex; PFC, prefrontal cortex; m-NMES, neuromuscular electrical stimulation on median nerve; LT, language training.

Figure 3 shows the detailed results of WA value in resting, LT and m-NMES-LT states. Compared with the resting state, WA values of the LT state showed significantly higher in contralesional PFC in the frequency intervals III (*p* = 0.009). In addition, m-NMES-LT state showed significantly higher WA values in contralesional PFC in intervals I (*p* = 0.05), II (*p* = 0.024), V (*p* = 0.05), and in ipsilesional MC in intervals I (*p* = 0.02), II (*p* = 0.011), III (*p* = 0.016), IV (*p* = 0.034), and V (*p* = 0.023) than the resting state.

### Functional connection analysis

**FIGURE 4:**
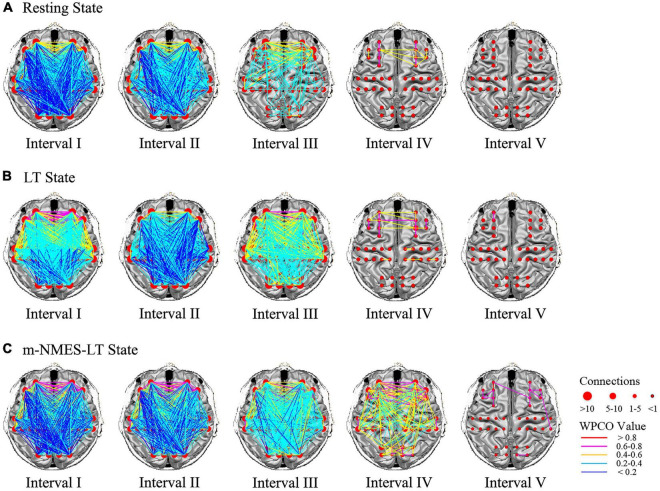
Frequency intervals I–V maps of the different state. The resting state **(A)**, LT state **(B)** and m-NMES-LT state **(C)**. Connectivity line indicates a significant wavelet phase coherence (WPCO) value between two channels. Line color indicates the connectivity intensity, and the sizes of the red dots indicate the numbers of connectivity among the channels. m-NMES, neuromuscular electrical stimulation on median nerve; LT, language training; WPCO, wavelet phase coherence.

Figure 4 shows the connectivity maps in the five frequency intervals under the different states. Visualized functional connectivity maps measured the degree of integration of brain networks. In this study, the results of significant differences between three states revealed interesting brain network characteristics. The connectivity was dense in intervals I to III and sparse in intervals IV and V; and increased connectivity was seen in intervals IV to V. In addition, the connectivity of the system regulatory activities did not change significantly in the three states, while the results of functional network changes in neurovascular coupling activities were all related to the prefrontal cortex.

**FIGURE 5:**
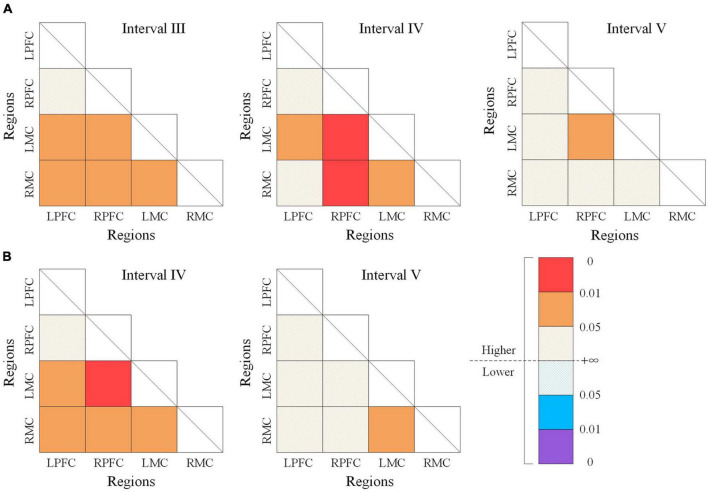
The wavelet phase coherence (WPCO) values of different state. Comparison of WPCO values between the resting state and m-NMES-LT state **(A)**, resting state and LT state **(B)**. Warm color represents the increased WPCO value; the brighter the color, the higher the significance. Cool color represents the decrease in WPCO value; and the deeper the color, the higher the significance. m-NMES, neuromuscular electrical stimulation on median nerve; LT, language training; WPCO, wavelet phase coherence.

The significant differences of WPCO values between the resting state and m-NMES-LT state as shown in Figure 5A. The WPCO value of m-NMES-LT state between ipsilesional PFC and ipsilesional MC in interval III (*p* = 0.037), IV (*p* = 0.026); ipsilesional PFC and contralesional MC in interval III (*p* = 0.042); contralesional PFC and contralesional MC in interval III (*p* = 0.044), IV (*p* = 0.006); contralesional PFC and ipsilesional MC in interval III (*p* = 0.017), IV (*p* = 0.001), and V (*p* = 0.043); ipsilesional MC and contralesional MC in intervals III (*p* = 0.042) and IV (*p* = 0.019) was significantly higher than resting state. Moreover, the WPCO of m-NMES-LT between ipsilesional PFC and ipsilesional MC in interval IV (*p* = 0.023); ipsilesional PFC and contralesional MC in interval IV (*p* = 0.04); contralesional PFC and ipsilesional MC in interval IV (*p* = 0.016); contralesional PFC and contralesional MC in interval IV (*p* = 0.016); ipsilesional MC and contralesional MC was significantly higher in interval IV (*p* = 0.024) and V (*p* = 0.044) than LT as shown in Figure 5B, and there was no significant difference in WPCO value was found between resting and LT states.

### Clinical validation

The behavioral comparison results after three consecutive weeks of treatment showed that although the WAB values of the two groups of patients showed an increase trend, the m-NMES-LT group had a greater increase, and the value of Repetition (*P* = 0.049) after treatment was significantly higher than before treatment, as shown as Table 3.

**TABLE 3:** Results of comparison of behavioral data before and after treatment: Mean (*SD*).

WAB	LT group		m-NMES-LT group	
		
	Pre/Post	*P-value*	Pre/Post	*P-value*
Spontaneous speech	2.45 (± 3.67)/5.09 (± 4.04)	0.125	2.90 (± 3.66)/6.40 (± 3.95)	0.055
Auditory comprehension	2.57 (± 2.12)/3.60 (± 2.15)	0.272	1.99 (± 2.24)/3.43 (± 1.95)	0.142
Repetition	2.11 (± 2.73)/3.0 (± 2.68)	0.449	0.89 (± 1.65)/2.78 (± 2.31)	0.049
Naming	1.10 (± 1.76)/1.65 (± 2.30)	0.539	0.84 (± 1.69)/1.81 (± 2.67)	0.345
Aphasia quotient	17.00 (± 17.29)/27.03 (± 20.32)	0.227	13.23 (± 16.17)/29.75 (± 19.98)	0.057

**FIGURE 6:**
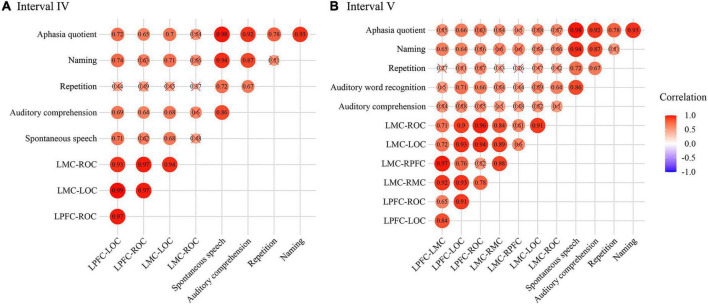
Correlation results of wavelet phase coherence (WPCO) and western aphasia battery (WAB) in frequency intervals III **(A)** and IV **(B)**. The intensity of the color and the diameter of the circle indicate the size of the correlation. Red means positive correlation and blue means negative correlation. WPCO, wavelet phase coherence; WAB, Western Aphasia Battery.

The results of Pearson’s correlation between WPCO and WAB showed that there was a significant positive correlation between WPCO value of LPFC-LOC and Spontaneous speech (*r* = 0.708, *p* = 0.011), Auditory comprehension (*r* = 0.685, *p* = 0.014), Naming (*r* = 0.742, *p* = 0.007), AQ (*r* = 0.715, *p* = 0.01), between WPCO value of LPFC-ROC and Spontaneous speech (*r* = 0.623, *p* = 0.027), Auditory comprehension (*r* = 0.638, *p* = 0.024), Naming (*r* = 0.628, *p* = 0.026), Aphasia quotient (*r* = 0.654, *p* = 0.02), between WPCO value of LMC-LOC Spontaneous speech (*r* = 0.676, *p* = 0.016), Auditory comprehension (*r* = 0.676, *p* = 0.016), Naming (*r* = 0.712, *p* = 0.01), Aphasia quotient (*r* = 0.695, *p* = 0.013), between WPCO value of LMC-ROC and Auditory comprehension (*r* = 0.596, *p* = 0.035), and Naming (*r* = 0.555, *p* = 0.048) in the frequency interval IV as shown in Figure 6A.

In frequency interval V, the significant positive correlation was founded between WPCO value of LPFC-LMC and Naming (*r* = 0.652, *p* = 0.02), between WPCO value of LMC-RMC and Naming (*r* = 0.604, *p* = 0.032), between WPCO value of LMC-RPFC and Naming (*r* = 0.597, *p* = 0.034), between WPCO value of LPFC-LOC and Spontaneous speech (*r* = 0.581, *p* = 0.039), Auditory comprehension (*r* = 0.709, *p* = 0.011), Naming (*r* = 0.638, *p* = 0.024), Aphasia quotient (*r* = 0.659, *p* = 0.019), between WPCO value of LMC-LOC and Auditory comprehension (*r* = 0.664, *p* = 0.018), AQ (*r* = 0.631, *p* = 0.025) as shown in Figure 6B.

## Discussion

This study mainly investigated the effects of two rehabilitation therapies (LT and m-NMES-LT) on the brain connectivity and clinical efficacy in patients with aphasia as measured by fNIRS. We found that the clinical efficacy of patients with m-NMES-LT for 3°weeks was better than LT through WAB score. In addition, m-NMES-LT could effectively increase the activation of the contralesional PFC and ipsilesional MC, and the WPCO of m-NMES-LT between ipsilesional and contralesional hemisphere was increased compared with LT. Specifically, we expected that the significant positive correlation between WPCO and WAB may contribute to a better understanding of the mechanism of m-NMES-LT in patients with aphasia.

Our previous study using fNIRS found that the distant cortical areas of stroke patients who received the m-NMES therapy produced functional reorganization. In particular, the neural coupling from the PFC to other brain areas was significantly increased in stroke patients following unilateral m-NMES. Some studies have shown that patients with aphasia exhibit changes in cerebral blood oxygenation and hemodynamics in the left PFC induced by language activities (Sakatani, 1998). Allendorfer JB et al. also reported that activation related to processing noun-verb semantic associations on the verb generation task were present in frontal, temporal and parietal regions. In addition, there was study reported that the m-NMES could improve the language function of coma patients. Therefore, we attempted to use m-NMES to treat stroke patients with aphasia on the basis of conventional language rehabilitation and use fNIRS to monitor their brain function, tried to observe the effect of m-NMES on language rehabilitation.

Five spontaneous oscillations with characteristic frequencies had been identified in fNIRS signals by using CWT in this study. We made further analyze for the data of acquired frequency intervals to obtain the WA which represents the changes of regional cerebral blood flow with the activity of cerebral cortex during different conditions. The WA results showed that the cerebral activation in frequency intervals I and II was significantly higher for m-NMES-LT state compared to that for resting state in contralesional PFC and ipsilesional MC. This result suggested that the cerebral blood flow in contralesional PFC and ipsilesional MC were changed during m-NMES-LT state. Because the oscillation in interval I and II mainly reflected the influence of respiratory and cardiac activities, which serve as pumps that drive blood through the vessels (Shiogai et al., 2010; Li et al., 2013). As a part of the systemic circulation, the effect of the cardiac and respiratory pumping is manifested in the vessels. This change was consistent with the theory that m-NMES may increase local cerebral blood flow and improve blood flow around hematoma (Liu et al., 2010). In addition, our results showed that compared with the resting state, WA values of the LT state had significantly higher in contralesional PFC in the frequency interval III. The m-NMES-LT state had significantly higher WA values in ipsilesional MC in intervals III and IV than the resting state. First, the oscillations in interval III were suggested to originate locally from the intrinsic myogenic activity of smooth muscle cells in resistance vessels. The vascular smooth muscles contracted or relaxed in response to an increase or a decrease in intravascular pressure respectively, and the myogenic mechanism might be partly under autonomic control. The oscillation in interval IV was a vascular reaction of neurogenic origin, which was closely related to frequency interval III. The continuous activity of the autonomous nervous system served to maintain the basal level of vasoconstriction. The nerves released substances that affect the smooth muscles activities, leading to changes in the vessel radius and resistance. The significantly increased WA in interval III and IV indicated an enhanced contractility of smooth muscle layer among contralesional PFC in LT, and an enhanced contractility of smooth muscle and neurovascular regulatory activity among ipsilesional MC in response to m-NMES-LT. The enhanced activity of the autonomous nervous system and the contractility of vascular smooth muscle caused by the nervous system are both significant for improving blood supply for blood vessels. Therefore, m-NMES-LT may play a role to the recovery of language function by improving the mechanism of blood supply to PFC and MC.

Another interesting finding was that the m-NMES-LT exhibited significant effect on spectral amplitude of cerebral oxygenation oscillations in frequency interval V. The oscillation in this interval reflected the influence of endothelial related metabolic activity. Metabolic regulation is the process of controlling blood flow according to the concentration of metabolites. Endothelial cells control the contraction and relaxation of smooth muscle by releasing vasodilators (such as nitric oxide) and vasoconstrictors to adjust the blood flow to satisfy the oxygen requirement of cells. Therefore, an increase in spontaneous oscillations could be the result of the endothelial layer activities. The neuroimaging studies about aphasia indicated if the reactivation and compensation of aphasia patients mainly occurred in the left hemisphere peri-lesional areas, resulting in relatively good recovery. In addition, evidence supported the communication between PFC and other brain regions, and this increased connectivity seemed to be crucial for therapy-induced improvements of aphasic word retrieval (Abel et al., 2015; Stefanie et al., 2015). In the present study, the significant increase of WPCO between ipsilesional PFC and ipsilesional MC in intervals V indicated that m-NMES-LT could enhance the functional connectivity of the left hemisphere in endothelial related metabolic activity. This might have important value for the rehabilitation of patients with aphasia.

It was worth noting that there was a significant positive correlation was found between WPCO associated with left hemisphere and WAB score, which confirmed that the increase of FC had a positive effect on the patient’s clinical performance. A number of studies had shown that higher complexity of functional connectivity is associated with better language performance post stroke (Yourganov et al., 2010), which was consistent with the findings of this study. This should be an important mechanism for the clinical efficacy of m-NMES-LT. In addition, previous studies had shown that NMES could enhance bilateral PFC activation (Huo et al., 2019), and both ipsilesional and contralesional PFC contribute to the recovery of aphasia (Belin et al., 1996; Harvey et al., 2017). The results of this study showed that although LT could induce the activation of PFC, the activation effect of m-NMES-LT seemed to be better. Therefore, we cautiously speculated that LT was more effective in activated PFC, which might be another mechanism of m-NMES-LT in clinical efficacy. These indicated that m-NMES-LT might have better clinical therapeutic effect than use LT.

Our study has several limitations. Firstly, although the sample size calculated was sufficient and the findings of this study are promising, the sample size was small, and, therefore, the study did not have sufficient power to provide firm conclusions. Future studies should recruit a larger sample size. Secondly, arterial pressure oscillations occurring spontaneously as Mayer wave existed in conscious participants in the vicinity of 0.1 Hz frequency, which may affect the FC in interval III (0.052–0.145 Hz). Therefore, the interference of Mayer waves should be considered in future studies. Moreover, the severity of patients was not classified. Different effects of m-NMES-LT on patients with mild and severe stroke were not analyzed in detail, and it is valuable to compare the change in brain function of different severity of stroke by applying fNIRS. The fNIRS is commonly used to detect the alternations of cortical functional plasticity. Limited by the detection depth, fNIRS cannot penetrate into the subcortical lesion such as basal ganglia region. More interesting results might be obtained by considering further refinement and investigation of the severity and classification of aphasia in subsequent studies.

The present findings revealed that m-NMES-LT could enhance cortical activation and brain functional connectivity in patients with aphasia, which was derived from myogenic, neurogenic, and endothelial cell metabolic activities. In addition, m-NMES-LT could improve clinical efficacy, and the increase of FC and the use of LT under the conditions of activation of regions contributed by m-NMES might be its possible mechanism of action. These suggested that m-NMES-LT might be more effective than LT in promoting clinical rehabilitation of patients with aphasia.

## Data availability statement

The raw data supporting the conclusions of this article will be made available by the authors, without undue reservation.

## Ethics statement

The studies involving human participants were reviewed and approved by Rehabilitation Center, Qilu Hospital of Shandong University and the Human Ethics Committee of National Research Center for Rehabilitation Technical Aids. The patients/participants provided their written informed consent to participate in this study.

## Author contributions

HX, ZL, and YW contributed to the conception and design of the study. HX, YM, and YS carried out the experiments. HX performed the data analyses and wrote the manuscript. HX and GX provided statistical assistance and support. JJ, JY, and XL provided opinions on grammar and rhetoric. All authors worked together to complete the manuscript, contributed to the manuscript revision, read, and approved the submitted version.
